# Delimiting a species’ geographic range using posterior sampling and computational geometry

**DOI:** 10.1038/s41598-019-45318-5

**Published:** 2019-06-20

**Authors:** Jonathan M. Keith, Daniel Spring, Tom Kompas

**Affiliations:** 1 School of Mathematics, Monash University, Clayton, Victoria 3800 Australia; 2 School of Ecosystem and Forest Sciences, The University of Melbourne, Melbourne, 3010 Australia; 3 Centre of Excellence for Biosecurity Risk Analysis, The University of Melbourne, Melbourne, 3010 Australia

**Keywords:** Biogeography, Conservation biology, Invasive species, Statistics

## Abstract

Accurate delimitation of the geographic range of a species is important for control of biological invasions, conservation of threatened species, and understanding species range dynamics under environmental change. However, estimating range boundaries is challenging because monitoring methods are imperfect, the area that might contain individuals is often incompletely surveyed, and species may have patchy distributions. In these circumstances, large areas can be surveyed without finding individuals despite occupancy extending beyond surveyed areas, resulting in underestimation of range limits. We developed a delimitation method that can be applied with imperfect survey data and patchy distributions. The approach is to construct polygons indicative of the geographic range of a species. Each polygon is associated with a specific probability such that each interior point of the polygon has at least that posterior probability of being interior to the true boundary according to a Bayesian model. The method uses the posterior distribution of latent quantities derived from an agent-based Bayesian model and calculates the posterior distribution of the range as a derived quantity from Markov chain Monte Carlo samples. An application of this method described here informed the Australian campaign to eradicate red imported fire ants (Solenopsis invicta).

## Introduction

Many of the questions arising in the management of threatened and invasive species require empirical estimation of geographic range limits and shifts in range limits over time. Delimiting surveys are routinely carried out as part of initial response to the discovery of an introduced species^1–3^ and to facilitate conservation efforts^4,5^, with management efforts focused within the delimited range. The effectiveness of programs to slow the spread of biological invasion depends upon accurate estimation of species range limits to avoid uncontrolled expansion of the invasion edge^6,7^. Accurate estimation of geographic range limits is also required for effective management of threatened species to ensure conservation efforts are applied to all locations where the species are present and to avoid costly actions being applied to unoccupied locations. The capacity to accurately estimate geographic range limits is also of central importance in understanding and predicting range shifts under environmental change to mitigate adverse impacts^8^.

Two related problems arise: design of efficient surveys and inference of boundaries. These problems are solved iteratively, in part because a species distribution evolves over time and in part because an inferred boundary informs subsequent monitoring efforts^9,10^. Here we focus on the inference problem, and present a new method that is applicable to a range of survey designs.

Yalcin and Leroux^11^ identify six methods for inferring a species’ range: observational study, grid-based mapping, convex hull, kriging, species distribution models and hybrid methods. They define an *observational study* somewhat idiosyncratically as a method that estimates a characteristic of a species range, such as the maximum elevation where a species can occur. *Grid-based mapping* and *convex hull* are methods for inferring a spatial distribution from a collection of point observations, and *kriging* is a method for interpolating spatial variables based on point observations and potentially also environmental covariates. *Species distribution models* estimate ranges based on correlations between species occurrence or abundance and environmental variables. *Hybrid methods*, as the name suggests, combine features of multiple types, for example pairing species distribution models with mechanistic modelling of spread processes.

For our present purpose we propose an alternative classification comprising four types of method: *utilization methods*, which characterize a species’ use of spatial resources based on detected individuals; *monitory methods*, which use records of survey actions (including those that did not result in detections) to delimit range; *correlative methods*, which identify correlations between environmental variables and occupancy or abundance of a species, and use these to infer where individuals may be present even if not observed; and *mechanistic methods*, which explicitly model spatial population dynamics and/or detection processes to identify plausible range distributions. These distinctions are primarily conceptual – advanced methods incorporate features from all of these categories.

### Utilization methods

One approach to range modelling involves *utilization distributions*. These provide a probabilistic representation of the use of spatial resources by an individual or species, across its range. Fleming *et al*.^12^ identify two distinct types: *range* and *occurrence* distributions. The range distribution “addresses the long-term area requirements of an animal, assuming its movement behaviors do not significantly change” whereas the occurrence distribution addresses the question of where the animal was located during the observation period. These definitions are framed in terms of an individual animal, but one can rephrase them for species in a straightforward manner. Methods for estimating range distributions include minimum convex polygon^13,14^, kernel density estimation^15^, mechanistic home range analysis^16^, autocorrelated Gaussian density estimation^17^, and local convex hull^18^. Occurrence distributions can be estimated using the Brownian bridge density estimator^19^.

Utilization methods model the internal structure of a spatial distribution. Here we focus on delimitation, that is, determining the limit of a species’ range and quantifying uncertainty in that limit. This is a challenging inference problem, and one that utilization distributions and their associated methods are not ideally suited to address. A common practice is to find a contour of the utilization distribution that encloses 95% of the observations^11^, but this, by definition, underestimates the extent of the range. The amount by which it underestimates is not apparent, and varies from one dataset to another.

Another problem for utilization methods is that available observations may not adequately represent the species’ range, for example due to a lack of sufficient monitoring resources, or imperfect detectability^20^. Consequently, even enclosing 100% of observations may exclude parts of the range where no observations were made. Prior to delimitation, it is typically not clear where monitoring is required. Moreover, there may be spatial variation in detection probability, due to environmental factors or to the use of multiple monitoring methods with different detection probabilities. To overcome this problem, it is necessary to model likely locations of undetected individuals, taking into account spatial variations in detection probability.

It may be possible to repurpose the delimitation method we present below to construct utilization distributions. However, we stress that utilization distributions are intended to characterize the *observed* use of spatial resources; they are not designed to represent the likely locations of unobserved individuals.

### Monitory methods

*Monitory methods* consider the history of survey actions undertaken during the management of a species, and combine detections, non-detections, and an assessment of detection probability to infer range limits, often by first constructing maps of probability of occupancy or expected abundance. For example, the method of Hauser *et al*.^14^ uses such records to construct a map of occupancy probabilities for an invasive plant species and prioritise subsequent survey actions.

Spatial variation in detection probability remains a problem for monitory methods, although in principle this spatial variation can be incorporated into the inference. An additional problem is that heat maps of probability of occupancy or expected abundance reflect both the geographic distribution of the species and uncertainty about the locations of undetected individuals. Consequently, a temporal sequence of such heat maps can create an illusion of range expansion merely due to increasing uncertainty regarding the locations of undetected individuals^7^, potentially even when the range within which detections occur is contracting. Boundary curves or polygons can be constructed by finding isopleths of such heat maps, but for any chosen threshold value, the resulting isopleth likewise reflects both the extent of the species’ range and the precision with which the available data delimit that range.

### Correlative methods

Correlative methods, known as *Species Distribution Models*^11,21^ *(SDMs)* involve regressing species occurrence or abundance against climatic or other environmental covariates, and then using maps of these covariates to predict the likely spatial distribution of undetected individuals. These methods work well when species are in equilibrium with their environment. However, this is unlikely to be true in many circumstances of management interest, because pest control programs typically are applied when species ranges are expanding, and threatened species programs often are applied when ranges are contracting. Moreover, SDMs typically do not take into account non-environmental biotic factors such as the presence or absence of diseases and predators.

*Ecological niche models*^22,23^ are also relevant to correlative methods. These characterize the distribution of a species in *environmental space* (also known as *ecological space*), in which points correspond to the values of a (potentially large) number of environmental or ecological variables. In contrast, *geographic space* is comprised of two-dimensional spatial locations. Typically, points in geographic space can be mapped to unique points in environmental space to assess whether they are suitable for a species, but this may be of little use if suitable habitats are unoccupied, as is often the case in invasion and conservation biology.

### Mechanistic methods

Another way to account for undetected individuals is to incorporate models of population dynamics into the inference procedure. In an invasive species context, the Bayesian approach developed by Mangel *et al*.^10^, estimates the probability of pest occupancy at different distances from the presumed invasion epicentre assuming the population expands smoothly, producing a bell-shaped spatial distribution. The delimitation method developed by Leung *et al*.^9^ was designed for invasions in which the proportion of invaded sites declines relatively smoothly from epicentre to edge.

The accuracy of these methods, which involve allocating survey effort along transects centered on the estimated invasion epicentre, is substantially reduced when individuals have a patchy distribution^9^. Boundary estimation for an expanding population can be challenging when spread occurs as a result of stratified diffusion^24^, in which individuals make frequent short movements and occasional long distance “jumps”. This form of spread process typically creates an irregular pattern of occupancy comprised of clusters of individuals. Clusters typically are located at imperfectly predictable distances from each other due to inherent difficulties in estimating the distances and directions of long distance movements^25^. This form of species distribution, which also can arise from spatial heterogeneity in habitat availability, creates a heightened risk of underestimating range boundaries because individuals may exist beyond the surveyed area despite an absence of detections near its perimeter. For contracting populations such as threatened species, range determination is complicated by complex source-sink dynamics^26,27^ that produce substantial gaps in occupancy. More generally, challenges in estimating range limits can arise when there is a complex interplay between species reproduction, dispersal rates and habitat suitability.

### An agent-based approach

In previous work^7^, we developed an agent-based model to reconstruct a history of the Brisbane fire ant invasion, or more precisely to sample multiple plausible histories from a posterior distribution using a Markov chain Monte Carlo (MCMC) technique. This approach combined features of all of the above methods. The available data included: extensive records of individual nest detection points, as in utilization methods; records of search actions and estimates of detection probabilities by targeted search and by public reporting in urban and rural environments, as in monitory methods; environmental variables in the form of a habitat suitability map, as in correlative methods; and a detailed model of population dynamics, including a distribution of founding distances, reproductive rate and a complete phylogenetic tree for all detected and putative undetected nests, as in mechanistic methods. While it is not possible to infer the exact number, locations or lifespans of undetected individuals, our method does simulate multiple plausible invasion histories at that level of detail. We typically sample 10000 such histories to explore the space of plausible histories consistent with the data. For the reader’s convenience, we provide a more detailed summary of the data and model parameters in Appendix 1. Full details of the model and the Markov chain Monte Carlo technique we used to sample from it are provided in Keith and Spring^7^, primarily in the Supplementary Information.

Our approach addressed many of the limitations identified above. In particular, it can be applied in circumstances where complex spatio-temporal dynamic processes create substantial gaps in occupied regions and irregular boundary shifts over time, using data obtained with imperfect and incomplete survey methods. However, one of our outputs involved processing the 10000 sampled histories to produce a time series of heat maps showing the expected areal abundance of fire ant nests. As we point out in our discussion of monitory methods above (and in our earlier paper), a time sequence of such heat maps can create an illusion of expansion due to increasing uncertainty regarding the location of undetected nests.

### Scope of this paper

Our goal in this paper is to provide a method for inferring and visualizing a species’ range limits given posterior sampled point sets, in such a way that the contribution of uncertainty to the apparent range is appropriately quantified. Each sampled point set includes known locations of detected individuals and putative locations of undetected individuals. In practice, we generate such point sets using our published agent-based method^7^. Next, we construct a polygon enclosing each point set, then identify map coordinates contained in the interior of at least a proportion *α* of these polygons. We provide boundaries for multiple values of *α* to indicate the degree of uncertainty in the inferred range.

The polygons are selected from a *polygon family*, thus constraining the polygons to have properties deemed desirable for a specific application, such as convexity or connectedness. In our examples we use *chi-shapes* - simple polygons constructed using an algorithm of Duckham *et al*.^28^ - or modified chi-shapes (newly proposed here) to allow for multiple disjoint polygons, as described in the section on Inferring Polygons below. Alternative polygon families could be used, for example to allow polygons with holes. To illustrate the new method we estimated the boundary of an invasive species that is subject to an eradication program. The method can also be readily applied to estimate boundaries of native species that are contracting or shifting due to environmental change, harvesting pressure or demographic variability. The program we consider is aimed at eradicating a fire ant invasion in South East Queensland, Australia. We estimated the boundary of the invasion at the end of April 2015, to inform a decision on whether to continue program funding, based on historical data regarding where fire ants were detected and where efforts were made to remove them. We compared our most conservative estimate to the operational boundaries in use by the eradication program at that time. We found that the outer operational boundary at the end of April 2015 (that is, the outer limits of the region monitored by remote sensing) corresponded over most of its length to our most conservative inferred boundary. On this basis, we concluded that the invasion had been successfully delimited, subject to modest extensions being made to the operational boundary in a few identified locations.

## Methods

The method takes as input multiple sets of points (that is, map locations) in a two-dimensional landscape, representing the locations of both detected and undetected individuals. These points may represent habitations, or alternatively the notional centre of range for each individual. Note that undetected individuals do not have known locations, and even the number of undetected individuals is unknown. Plausible locations for undetected individuals must therefore be imputed via some algorithm. We assume that multiple alternative sets of points are available, each containing locations of all detected individuals, but differing in the number and locations of imputed undetected individuals.

In principle, such sets of points do not have to be generated within a Bayesian framework: any algorithm capable of imputing missing data will suffice. However, the probabilistic interpretation that we give to the polygons constructed here assumes that the multiple sets of points have been sampled from a posterior distribution. In the examples presented below we use an MCMC algorithm that we developed^7^ to sample from a posterior distribution over plausible histories of a biological invasion.

### Input to the method

The input consists of the following items:

1. Point sets *P*_1_, *P*_2_, …, *P*_*N*_, where each *P*_*i*_ contains *n*_*i*_ two-dimensional points.
2. A set *Q* of reference points distributed throughout the region of interest.
3. A value *α*, such that the polygon to be constructed will contain all reference points interior to at least a proportion *α* of the *N* polygons constructed for the *N* point sets (see Step 1 in the next section).
4. A family of polygons ${\mathcal F}$ and a map ℘ such that any set of points *P* maps to a unique polygon $\wp (P)\in  {\mathcal F}$. In this paper, all polygons are chi-shapes (defined below) or modified chi-shapes.

Each of the point sets *P*_1_, *P*_2_, …, *P*_*N*_ includes a subset of observations common to them all, representing known locations of individuals. The point sets differ in the number and locations of undetected individuals, imputed by some appropriate method. Here we use the posterior sampling method of Keith and Spring^7^.

The set *Q* of reference points provides a convenient discretization of the geographic region of interest. In principle it can be any collection of points scattered throughout the region, but in this paper we use the centres of cells in a square tiling. In that case, the locations of all reference points can be determined by supplying map coordinates of one reference point (in some specified coordinate system aligned to the tiling) and the side length of the tiling.

The value *α* controls how confident we can be that the polygon we ultimately report contains the *entire* range of the species. We stress that neither *α* nor 1 − *α* should be interpreted as a proportion of the range of the species. Whatever value of *α* is used, the resulting polygon will contain all known locations of individuals, since these are common to all point sets, and thus contains the entire *observed range* of the species. But our goal is to construct a polygon that also contains all unobserved members of the species, and *α* reflects how conservative we want to be in constructing such a polygon.

Various options are available for the family of polygons ${\mathcal F}$. One simple choice is the family of convex polygons, in which case ℘(P) would be the convex hull of a set of points *P*. However, convex polygons have the disadvantage of resulting in potentially substantial overestimation of the species boundary when actual boundaries are nonconvex. Nonconvex boundaries are likely in many circumstances, including where unsuitable habitat prevents areas being occupied and where long-distance movements cause the boundary to “bulge outwards” in the vicinity of satellite populations.

**Figure 1:**
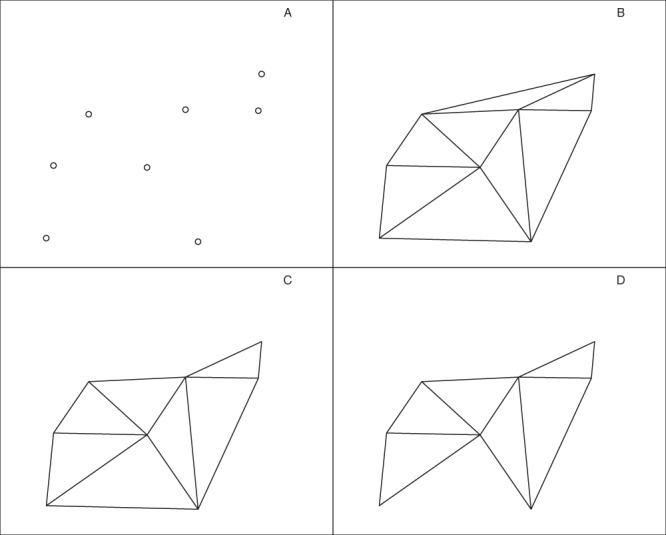
Duckham’s Algorithm for constructing chi-shapes. (**A**) A point set. (**B**) The corresponding Delaunay triangulation. The external edges form the polygonal boundary of the convex hull of the point set. (**C**) The chi-shape obtained by removing the longest external edge of B, thus creating two new external edges and one new external vertex. The external edges of C now form a polygon that is no longer convex, but remains simple. (**D**) The chi-shape obtained by removing the longest external edge of C. The longest external edge of D cannot now be removed because the external edges of the remaining triangles would not form a simple polygon. Note the longest external edge of D can be removed according to our modified criterion (2′).

*Chi-shapes*^28^ are a family of simple polygons (‘simple’ in the geometric sense that sides intersect only at corners, and form a closed path). This family includes all convex polygons, but chi-shapes may also be non-convex. A chi-shape ℘(P) is constructed for a set of points *P* by starting with the Delaunay triangulation of *P*, then identifying all external edges that satisfy two criteria: (1) the edge is longer than a given length *L*; and (2) if the edge is removed, the external edges of the remaining triangles still form a simple polygon. Only the longest such edge is removed, necessarily creating two new external edges and one new external vertex. This process is iterated until no external edges satisfying these criteria remain (see Fig. 1).

**Figure 2:**
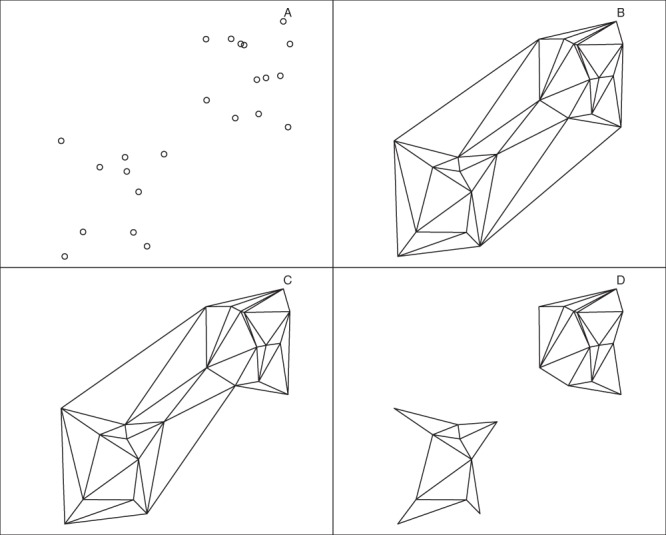
Constructing modified chi-shapes. (**A**) A point set. (**B**) The corresponding Delaunay triangulation. (**C**) The chi-shape obtained by removing the longest external edge of (**B**). The remaining triangles include all vertices. (**D**) The chi-shape obtained by iteratively removing the longest external edge, along with any other external edges in the same triangle, until two disjoint components are formed.

In this paper, all polygons are either chi-shapes or modified chi-shapes in which we relax criterion (2). We proceed as in the preceding paragraph, except that we replace criterion (2) with the requirement: (2′) if the edge is removed, along with any other external edges in the same triangle, the remaining triangles still include all vertices (see Fig. 2). The properties of this algorithm should be analysed in future work; here we merely note that by removing the other external edges in the same triangle, it becomes possible to form disjoint polygons.

Numerous other polygon families are available, for example, the families of polygons produced by LoCoH^18^ or by parametric kernel density estimation^29^. We do not claim that chi-shapes or modified chi-shapes are preferable to these alternatives; a comparison is a potential direction for future research.

### Inferring boundaries

Our proposed method consists of the following steps:

1. Construct a polygon ${\wp }_{i}=\wp ({P}_{i})\,\in  {\mathcal F}$ for each point set *P*_*i*_.
2. For each reference point, count the number of point sets for which the polygons constructed in Step 1 contain that reference point in their interior or on their edge.
3. Identify the set of reference points ${Q}_{\alpha }\subset Q$ for which the counts determined in Step 2 exceed a proportion *α* of the total number of point sets *N*.
4. Construct a polygon $\wp =\wp ({Q}_{\alpha })\,\in  {\mathcal F}$ using the reference points identified in Step 3.

If a high resolution is desired, the number of reference points may be large. In that case, Step 4 can be computationally intensive. The computational efficiency of Step 4 can be improved if the reference points are centres of cells in a square tiling, as in all our examples below. In that case, we can first identify *boundary reference points*. A reference point in a square tiling is said to be on the boundary if any of the four reference points immediately above, below, to the left or to the right of the point is contained in fewer than a proportion *α* of the polygons constructed in Step 2. Step 4 then consists of constructing a polygon only for these boundary reference points. If the polygons are chi-shapes or modified chi-shapes, and the length *L* used in their construction is sufficiently large relative to the spacing between reference points, the polygon will be the same as if all of the reference points identified at Step 3 had been used.

**Figure 3:**
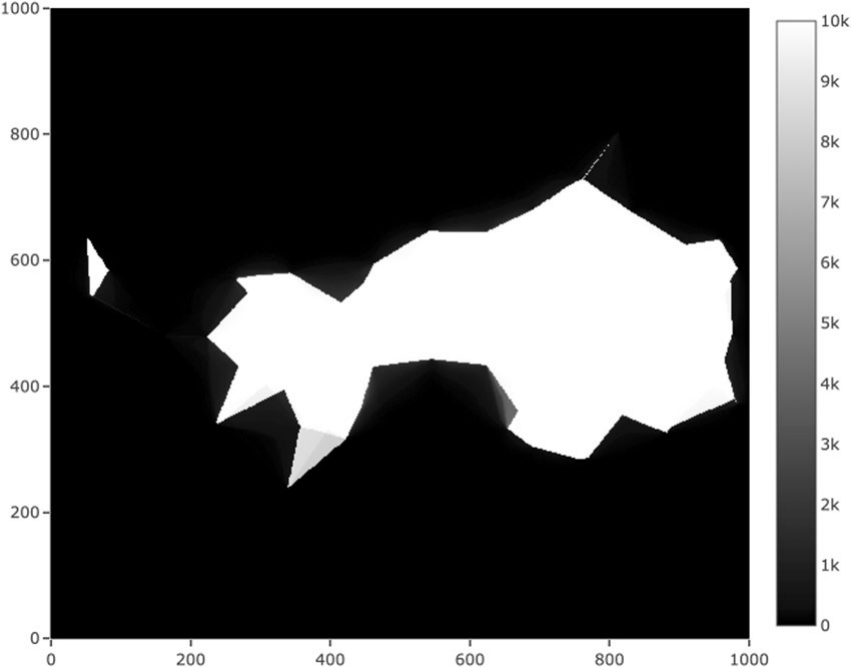
An example of a heatmap indicating the number of point sets (out of 10000) for which the corresponding chi-shape contains each reference point. Reference points are at the centres of cells of side length 100 m in a square tiling. There are 1000000 reference points covering a 100 km by 100 km region. The values shown in this heat map were used to construct the polygons in the December 2014 subplot of Fig. 7.

Using the centres of a square tiling as reference points also facilitates an alternative visualization. The counts obtained at Step 2 (or alternatively the proportions obtained by dividing these counts by *N*) form a data matrix that can be visualized using a heat map. This heat map is of interest in its own right, and we present an example below (Fig. 3). One can also replace Steps 3 and 4 above with an algorithm for tracing an isopleth of the heat map, that is, the level set corresponding to the level *α*. However, in that case the resulting polygon may not belong to the desired polygon family ${\mathcal F}$.

The proposed method can be interpreted as averaging classifiers built from multiple point sets. That is, one can interpret the polygons built at Step 1 as classifying space into infested regions (interior) and non-infested regions (exterior), with the above-mentioned heat map being essentially an average of these classifiers. In this respect, our method resembles *range bagging*^30^. The resemblance is somewhat superficial however, as range bagging is primarily a computational technique for building classifiers in high-dimensional environmental space by averaging over classifiers in one or two dimensions. Moreover, range-bagging generates multiple point sets via sub-sampling observations rather than by imputing locations to undetected individuals.

For the analysis presented below, we experiment with square tilings having spacings of 50 m and 100 m. We also experiment with setting the minimum length of edges to be removed in the construction of chi-shapes and modified chi-shapes to be *L* = 5 km, 10 km and 20 km.

## Results

### Simulation study

To test the capacity of the method to infer the geographic range of a species, and in particular to quantify the likely locations of undetected individuals, we used a simulated data set that we had previously generated to mimic a biological invasion and eradication program^7^. The simulation involved constructing an entire detailed history of a hypothetical invasion, starting with an initial introduction, recording individual founding events, including time of founding and location of all individuals, and also simulating management efforts to identify which individuals were detected and thus available for inference, and which nests were killed by treatment. Further details of the simulated invasion and our reconstruction of it are provided in Keith and Spring^7^ and are summarised in Appendix 2 below for the reader’s convenience. Here the relevant points are the following:

1. We sampled 10000 plausible histories of the invasion from a posterior distribution. From each of these we extracted the known (for detected nests) and imputed (for undetected nests) locations of all individuals alive during the second last month of the modeled period. We chose the second last month so that the imputed locations of undetected individuals would be informed by detections made in the final month. This produced 10000 point sets.
2. Because the data is simulated, we also know the true history of the invasion, including the precise location and lifespan of all detected and undetected individuals. From this we extracted the true locations of all individuals alive during the second last month of the modeled period.

**Figure 4:**
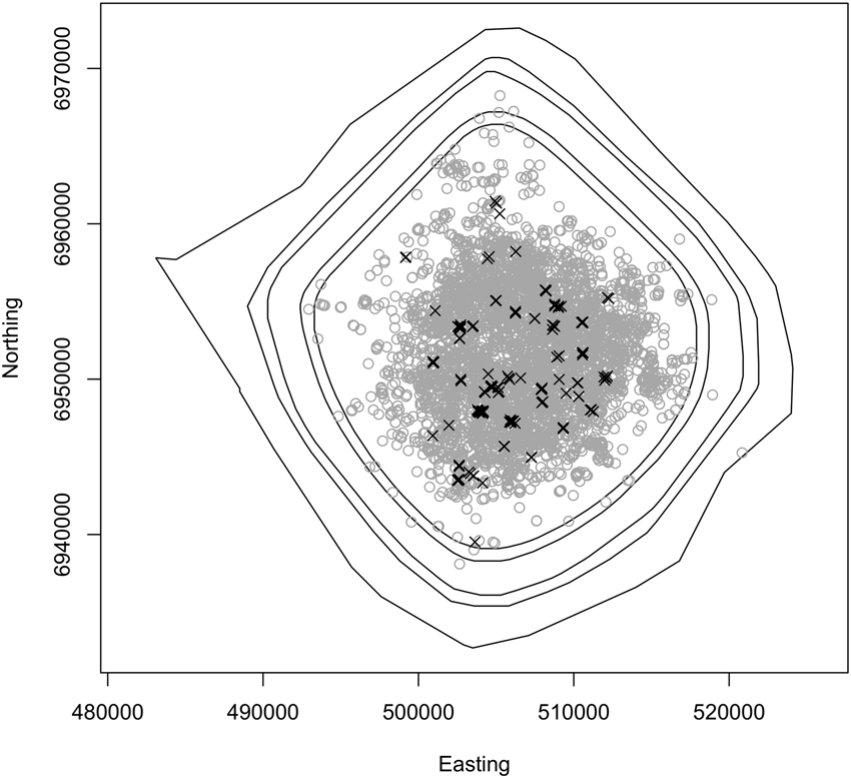
Inferred boundaries of the simulated invasion in the second last month of the modelled period. The five boundaries correspond to 1 − *α* = 0.5, 0.75, 0.975, 0.99 and 0.999 (innermost to outermost). True locations of all individuals alive in the second last month are shown as grey circles, and the locations of detections that occurred during that month are shown as black crosses.

Figure 4 shows inferred boundaries for 1 − *α* = 0.5, 0.75, 0.975, 0.99 and 0.999 (innermost to outermost). Note that here and in the rest of the paper we specify values of 1 − *α*, rather than *α*, purely for the aesthetic reason that the area enclosed increases as 1 − *α* increases.

Figure 4 also shows the true locations of all individuals that were alive in the second last month of the period modeled, and the locations of detections that occurred during that month. Note that all of the detections are inside the 0.5 boundary. Indeed, they must be contained in the boundary inferred for any value of *α*, since they are contained in all 10000 point sets.

### Case study: fire ants in brisbane

The method presented here was developed for the National Fire Ant Eradication Program (NFAEP) to eradicate the Red Imported Fire Ant (RIFA) from the vicinity of Brisbane, Australia. As the history of this eradication program underscores the importance of accurately delimiting an invasion, we provide the following summary.

During the early years of the NFAEP, control efforts were focused primarily on known infestations and nearby areas, with relatively little surveillance around those areas. This strategy can be slow in achieving delimitation when infestations exist well beyond the boundary of the managed area. Infestations that were accurately delimited in the early years of the program, such as the Fisherman’s Island infestation, were successfully eradicated^31^, while infestations that were not accurately delimited have continued to spread. In June 2007, RIFA colonies were detected at Amberley in Brisbane’s southwest, outside the operational area at that time. It was subsequently determined that an invasion had been spreading undetected from a point in or near Amberley for an extended period. This realization was a major setback for the eradication program, which had been operating with apparent success since 2001. In previous modeling^7^, we estimated that eradication was close to being achieved by 2004, but that the population subsequently recovered, in large part due to delimitation failure. Our results indicated that Amberley was not the only delimitation failure – there were undetected areas of spread in the eastern part of the invasion at around the same time, and these contributed to the recovery after 2004.

Due to continuing spread of the Australian fire ant invasion, the eradication program’s funding and methods were reviewed. It was decided that continued funding of the program beyond June 2013 would depend partly on the invasion being successfully delimited by 30 June 2015. To increase confidence that delimitation had been achieved, the NFAEP surveyed a large area near the invasion’s estimated boundary in 2013 and 2014. To undertake this task, low cost monitoring methods involving remote sensing and citizen monitoring were applied. These methods have substantially lower detection probabilities than conventional surveillance methods, including ground surveillance with trained personnel, but enable large areas to be rapidly surveyed at affordable cost. This reliance on a surveillance method with detection probability substantially less than 1 highlights the importance of accounting for this source of observational error in estimating the invasion’s boundary.

At the time this analysis was performed, we had data on detections and interventions to the end of May 2015. We decided to assess whether the invasion had successfully been delimited by the end of April 2015, so that the inference would be informed by one month of subsequent detections. We first inferred a complete history of the invasion using a Bayesian agent-based model previously developed for reconstructing the Brisbane RIFA invasion^7^ and summarized in Appendix 1.

The remote sensing efficacy (ie. probability of a nest being detected by aerial survey) and the founding rate (ie. average number of nests founded per nest per month) were held fixed rather than inferred, but we investigated the impact of alternative fixed values on inferred boundaries. We therefore performed five separate MCMC runs with:

1. Remote sensing efficacy 0.2, founding rate 0.25 nests founded per nest per month.
2. Remote sensing efficacy 0.3, founding rate 0.25 nests founded per nest per month.
3. Remote sensing efficacy 0.4, founding rate 0.25 nests founded per nest per month.
4. Remote sensing efficacy 0.3, founding rate 0.15 nests founded per nest per month.
5. Remote sensing efficacy 0.3, founding rate 0.35 nests founded per nest per month.

The values of remote sensing efficacy and founding rate selected for these runs reflect ranges of plausible values for these parameters, according to advice received from Biosecurity Queensland.

Each run was continued until at least 40000 MCMC reconstructed histories were produced, with the first 20000 discarded as burn-in. Convergence was assessed visually using time-series plots of log-likelihood. For each of the reconstructed histories, we extracted the map coordinates of nests living at the end of April 2015. Thus each of our inferred boundaries was based on at least 20000 point sets.

**Figure 5:**
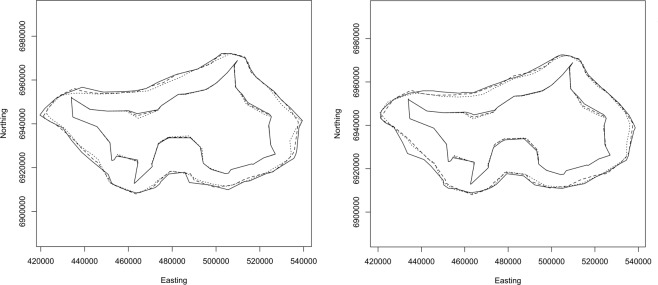
(Left) Inferred 50% (inner group) and 99.9% (outer group) boundaries at the end of April 2015. Results are shown for founding rates of 0.15 (dotted), 0.25 (dashed) and 0.35 (solid) nests per nest per month. All results are for a remote sensing efficacy of 0.3. (Right) Inferred 50% (inner group) and 99.9% (outer group) boundaries at the end of April 2015. Results are shown for remote sensing efficacies of 0.4 (dotted), 0.3 (dashed) and 0.2 (solid) nests per nest per month. All results are for a founding rate of 0.25 nests per nest per month.

Figure 5 (left) illustrates the 0.5 (inner group) and 0.999 (outer group) boundaries for the three runs with assumed remote sensing efficacy 0.3, and founding rates 0.15, 0.25 and 0.35 nests per nest per month. As expected, the inferred geographic extent increases with the founding rate. However, the difference is negligible for the 0.5 boundaries, and not large even for the 0.999 boundaries. We will therefore ignore the influence of founding rate and use the middle founding rate of 0.25 nests per nest per month (that is, 3 nests per nest per year) in the analyses that follow.

Figure 5 (right) shows the 0.5 (inner group) and 0.999 (outer group) boundaries for the three runs with assumed founding rate of 0.25 nests per nest per month, and remote sensing efficacies of 0.2, 0.3 and 0.4. As expected, the inferred geographic extent increases as the assumed remote sensing efficacy decreases, but again the difference is negligible, and we will use the middle remote sensing efficacy of 0.3 in the remaining analysis. It should be noted that finding that the value we assume for remote sensing efficacy has little effect on the inference is completely different to saying the success of the program does not depend on the actual value. This is because the inference is informed by multiple data types, so that the past can be accurately reconstructed even without a precise estimate of remote sensing efficacy. Nevertheless, the eventual success of the program may depend crucially on rapid detection of relatively rare long-distance dispersal events by remote sensing.

**Figure 6:**
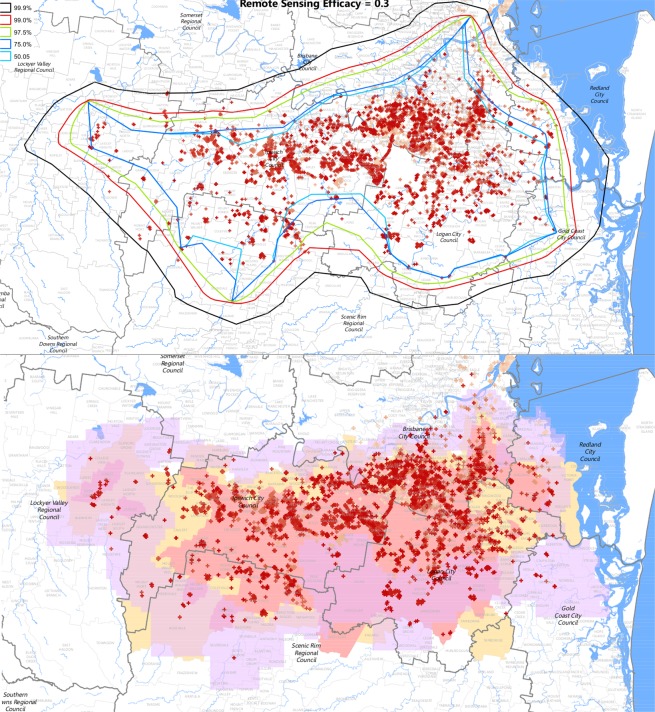
(Top) Inferred 50%, 75%, 97.5%, 99% and 99.9% boundaries at the end of April 2015. Remote sensing detection probability was set to 0.3 and founding rate was set to 0.25 nests per nest per month. (Bottom) Low-risk restricted areas (in yellow), high-risk restricted areas (pink) and remote sensing scope (purple). Also shown in both maps are crosses marking previous detection points as at 8 July 2015, colour coded by time of detection, with the most recent detections in red and the oldest in pale brown. (Figure created with the assistance of Bob Bell of Biosecurity Queensland using ArcMap 10: www.esri.com).

Figure 6 presents our main result – inferred 0.5, 0.75, 0.975, 0.99 and 0.999 boundaries at the end of April 2015 assuming a founding rate of 0.25 nests per nest per month and remote sensing efficacy of 0.3. This figure also shows the operational boundaries in place at that time. These included a region designated the remote sensing scope, and low- and high-risk restricted areas. The remote sensing scope is a region that is monitored by airborne cameras. However, only a small part of this area is searched in any one month. The restricted areas have various management strategies in place to limit human-assisted movement of RIFA and to eradicate existing infestations.

### Delimitation in time-series

**Figure 7:**
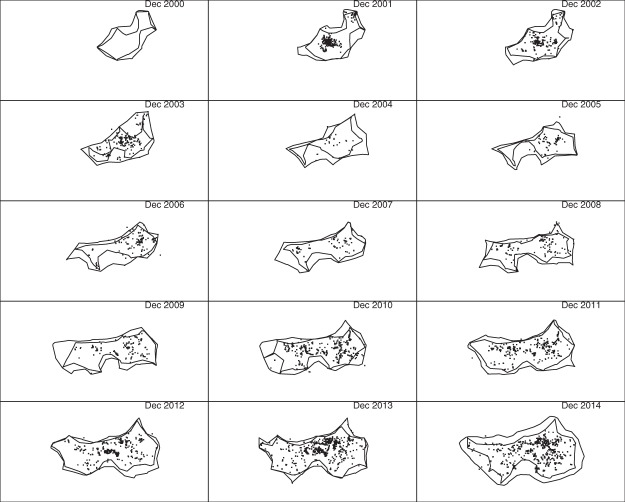
The 15 sub-plots represent the geographic extent of the Brisbane fire ant invasion in December 2000–2014. Chi-shapes enclose reference points contained in at least 5000 (inner polygon) and 9990 (outer polygon) of the 10000 chi-shapes for individual point sets. Reference points are centres of 100 m by 100 m cells in a square tiling. Nests detected throughout the year are shown as small points. The minimum length of edges removed in the construction of chi-shapes was *L* = 10 km.

One reason for proposing the delimitation method presented here was dissatisfaction with using our earlier abundance heat map^7^ to delimit boundaries, given its tendency to exaggerate apparent spatial extent due to uncertainty regarding the location of undetected individuals. This effect is most apparent when visualizing changes in boundaries over time, since uncertainty about the location of undetected nests tends to increase towards the end of the data collection period. Figure 7 shows the 0.5 (inner) and 0.999 (outer) inferred boundaries in December 2000–2014, using chi-shapes with *L* = 10 km and a square tiling with cells of 100 m by 100 m. Also shown are all detections that occurred January–December of each year (some of which are outside the December boundaries, due to clearing the pest from those areas earlier in the year).

We propose that the series of 0.5 polygons gives the best visual representation of temporal change in boundary location, since these polygons are somewhat analogous to medians, and thus less affected by increasing uncertainty. On the other hand, if one wants to identify a region that contains the entire infestation with high probability, we recommend the 0.999 polygon. The gap between these two polygons gives an indication of the degree of uncertainty in boundary location, and spatial variation in that uncertainty. Note this gap is wider in the December 2014 plot than at earlier times, but otherwise fairly constant.

The December 2000 subplot illustrates one of the advantages of our approach: it shows the inferred extent of the infestation prior to the first detections in 2001. This is possible because our sampling algorithm^7^ imputes plausible histories, including time of founding, for all nests. Similarly, the infestation centred on Amberley is visible in the west in December 2004 and 2005, even though no detections occurred there in those years.

To investigate the effect of changing the spacing between reference points, we also produced results using 50 m by 50 m cells. The results (not shown) were visually indistinguishable from Fig. 7. We concluded that our method is not much affected by cell size, at least when the side length of cells is small compared to the parameter *L*.

### Modified chi-shapes

**Figure 8:**
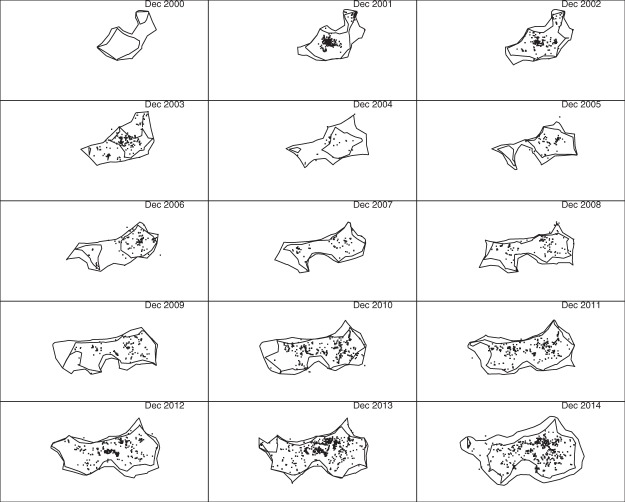
The same point sets used to construct the chi-shapes in Fig. 7 were used here to construct modified chi-shapes. As in Fig. 7, 100 m by 100 m cells are used in the square tiling, and the minimum length of removed edges was *L* = 10 km. The only difference is that criterion (2′) was used instead of criterion (2) to decide whether an edge could be removed. (See main text for explanations of these criteria).

Figure 8 shows similar results using modified chi-shapes, with all other settings the same. The advantage is that inferred boundaries can separate into disjoint polygons. This occurred for some of the 0.5 polygons, but none of the 0.999 polygons. In particular, one can see that there were two disjoint infestations in 2000, consistent with two separate introductions. The Amberley infestation can also be seen spreading separately from the main infestation between 2003 and 2006.

**Figure 9:**
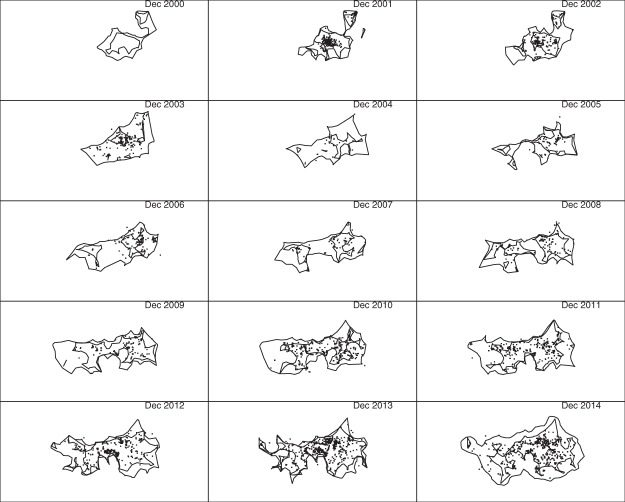
The algorithm used to construct Fig. 8 was repeated with *L* = 5 km. All other data and parameters were the same.

**Figure 10:**
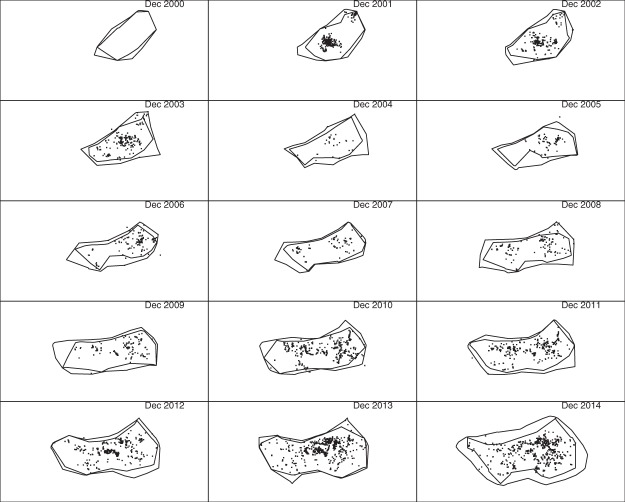
The algorithm used to construct Figs 8 and 9 was repeated with *L* = 20 km. All other data and parameters were the same.

To investigate the effect of varying the parameter *L* used in the construction of chi-shapes and modified chi-shapes, we repeated the analysis with *L* = 5 km and *L* = 20 km (Figs 9 and 10). The shape of the 0.5 polygons is substantially affected by the choice of *L*: with *L* = 5 km these polygons fragment into multiple disjoint components, whereas with *L* = 20 km only a single connected polygon is produced. The 0.999 polygons are much less affected by this parameter: all 0.999 polygons remained connected for all three values of *L*, although they do become increasingly “rough” as *L* decreases.

Changing the parameter *L* has a less dramatic effect on the 0.5 polygons when chi-shapes are used instead of our modified chi-shapes, because chi-shapes are constrained to be simple polygons.

For management actions that rely on containing the infestation with high probability, such as setting the limits of aerial searches, the 0.999 polygons will be of more interest than the 0.5 polygons. In that case, the appropriate choice of *L* is a less pressing concern. However, efficient allocation of resources within the boundary may be better guided using an abundance or occupancy heat map, given the sensitivity of the 0.5 polygons to the choice of *L*.

### Comparison to utilization methods

The method for estimating range limits described in this paper is unique in basing the inference on multiple sets of imputed coordinates representing locations of undetected individuals. It thus addresses a fundamentally different problem than utilization approaches. Both approaches identify spatial distributions, but those produced by utilization approaches represent a species’ observed use of spatial resources, whereas those produced by the new method represent posterior uncertainty in the location of range limits, accounting for undetected individuals. Nevertheless, it is interesting to compare our results to utilization approaches.

**Figure 11:**
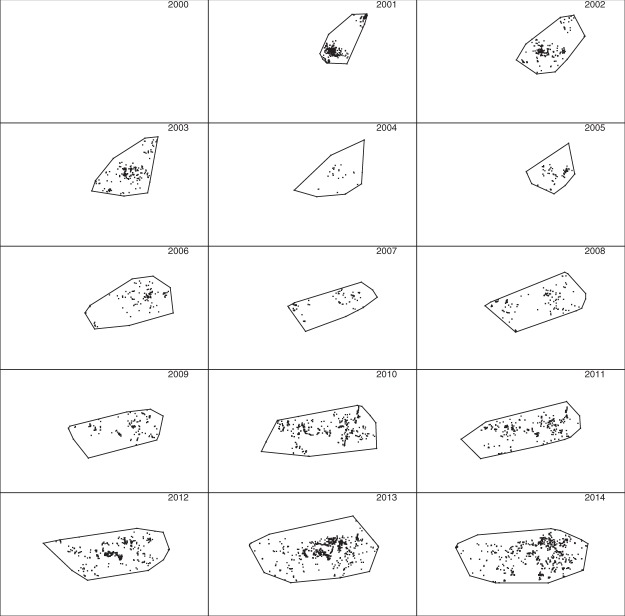
The 15 sub-plots represent the geographic extent of the Brisbane fire ant invasion in 2000–2014. Note each sub-plot represents an entire year, not the month of December as in Figs 7–10. Polygons are convex hulls for all nests detected in the corresponding year. Detected nests are shown as small points.

**Figure 12:**
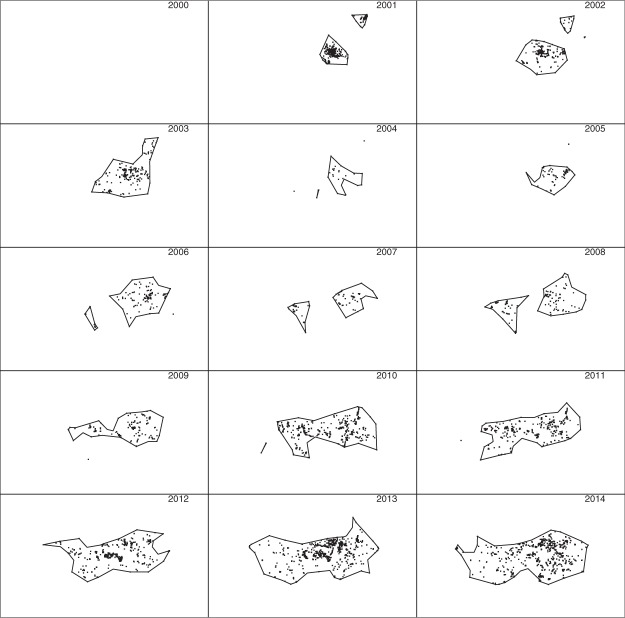
The 15 sub-plots represent the same observations as Fig. 11, but with polygons constructed using the r-LoCoH method instead of convex hull.

We constructed polygons using detections made in each of the years 2001–2014, using two approaches: convex hull (Fig. 11) and the r-LoCoH method^32^ with *r* = 10 km (Fig. 12). The parameter *r* is the maximum distance of neighbors used to construct a local convex hull around each detection.

Note that we used only detections, not imputed locations of undetected nests, in this analysis, to highlight the advantage of using posterior sampling to impute locations of undetected nests. As noted above, it would also be possible to use LoCoH polygons in place of chi-shapes at Steps 1 and 4 of our algorithm, but we have not explored this possibility.

The first subplots of Figs 11 and 12 are blank because there were no detections in 2000, which in itself highlights an advantage of posterior sampling of unknown locations and founding times: inferences can be made about species distribution at times prior to the first detection. In the subplots for later years, polygons constructed using only detections do not identify several large infested regions inferred using our method. For example, compare the western infestations shown in the 2004 and 2005 sub-plots of Fig. 7 to the corresponding sub-plots in Figs 11 and 12. These regions are not apparent using either convex hull or r-LoCoH, mainly because large infested areas went undetected in those years. Our inference for those years is informed by detections made prior to 2004 and subsequent to 2005, and by models of unobserved spread. The convex hull approach also demonstrates the opposite problem – the convexity of the polygons forces inclusion of large regions that are clearly not infested. For example, compare subplots for the years 2006–2009: a large concave region is apparent in the south in Fig. 7, but not in Fig. 11. Also note that the polygons shown in Figs 11 and 12 enclose all detections from the corresponding year; had we used only the detections made in December of each year, these polygons would have been much smaller and would have failed to enclose large infested regions. Thus the temporal resolution possible with our method is much higher.

Another advantage of our method is that by constructing polygons for multiple values of *α*, one can visualize the uncertainty regarding boundary location, and spatial variation in that uncertainty. While it would also be possible to construct multiple polygons enclosing different proportions of the detections, these would reflect relative utilization of regions internal to the boundary, not uncertainty regarding the boundary location.

## Discussion

The method presented here constructs simple connected polygons representing the boundary of a species’ geographic range. The simulation results shown in Fig. 4 demonstrate that boundaries constructed using the proposed method do indeed reflect the location of actual nests, including undetected nests. Note that the detections made in the month for which these boundaries were constructed do not provide a good indication of the actual range of the species: if only these detections were used to infer the boundary the range would be severely underestimated. Also note that by constructing boundaries for different values of *α*, a realistic indication of the uncertainty in the location of the boundary can be obtained. Most living individuals are contained within the 0.5 boundary, and all but one of the undetected individuals are contained within the 0.975 boundary, with the remaining individual between the 0.99 and 0.999 boundaries.

The meaning of the value 1 − *α* requires some clarification. Strictly speaking, for each reference point contained within the 1 − *α* boundary, *α* is the proportion of point sets for which the corresponding polygon contains that reference point. If the point sets are sampled from a posterior distribution, and the shape of the species’ range is well approximated by a member of the polygon family, the 1 − *α* boundary can be interpreted as containing all points with a posterior probability at least *α* of being within the geographic range of the species.

Importantly, the polygons constructed by this method are not required to be convex, giving the method greater generality and flexibility than previously applied convex polygon methods^14^. Figure 6 illustrates that boundaries of real species distributions can be concave, and would not be well approximated if the polygon were constrained to be convex. This is most noticeable along the northern boundary, where use of a convex polygon would unnecessarily include a large geographical area within the inferred range. This demonstrates the risk of overestimating the boundary when convex polygon methods are used. Species often have nonconvex distributions resulting from spatial variation in habitat suitability and long-distance dispersal events that create outlier populations in remote locations.

For the fire ant data, we found that the extent of the invasion was likely to be within operational boundaries at the end of April 2015, with the outer edge of the area remotely sensed corresponding over most of its length to the outer edge of the 0.999 inferred boundary. On this basis, we concluded that the invasion had been accurately delimited by the end of April 2015, subject to small extensions to operational boundaries in the southeast, far west and north of the Brisbane River, near the coast. Founding events rarely occur across large bodies of water. This behaviour is not incorporated into our model, so our methods may overestimate expansion north of the river. While this does not guarantee that eradication will ultimately be achieved, or that delimitation failure will not recur at some time in the future, establishing that the invasion has been delimited is an essential prerequisite to the ultimate success of the program.

The approach developed here is well suited to practical applications for assisting managers of biological invasions and threatened species. Invasion management effectiveness can benefit from the capacity to regularly update estimates of the invasion boundary whenever new information is obtained during the course of an eradication or containment program. Such information is vital to determine whether management efforts are succeeding in contracting the invasion or slowing its spread. Regular updating of range limits also is required to assess whether threatened species populations that are subject to management are expanding or not contracting.

Our method of constructing polygons is not limited to posterior samples obtained using MCMC. For example, it could alternatively be used with posterior samples obtained using Approximate Bayesian Computation (ABC – see the seminal paper of Beaumont *et al*.^33^ for a description). Our method requires multiple alternative point sets representing plausible locations of individual entities, but these need not even be generated via posterior sampling if alternative means of imputing missing locations are devised.

Although in this paper we have focused on the computational geometry aspects of the method, the usefulness of the resulting polygons depends crucially on the posterior sampled point sets, which we generated using our earlier agent-based Bayesian approach^7^. The agent based approach draws together components of utilization, monitory, correlative and mechanistic approaches, and takes into account the species’ life cycle, environmental variables and human interventions. It is a highly flexible approach that can potentially be modified for a wide variety of species, and could also incorporate genetic information, thus refining estimates of population dynamic processes and increasing the accuracy of estimated range limits.

The wide range of potential application of our approach will allow it to make substantial contributions to the problems posed by biological invasions and conservation of threatened species.

An R package *pts2polys* implementing the method described herein is available from CRAN. Currently this package uses chi-shapes, but not the modified chi-shapes we introduced above. C code implementing the method for modified chi-shapes is available from https://github.com/jonathanmkeith/posterior_polygons/releases/tag/v1.0.

## Supplementary information

Delimiting a species’ geographic range using posterior sampling and computational geometry (Appendices)

## Data Availability

Data and code used in this paper are available on request to the corresponding author.
