# Supplementary material for: Delimiting a species’ geographic range using posterior sampling and computational geometry
Source: Sci Rep. 2019 Jun 20;9:8938. doi: 10.1038/s41598-019-45318-5 (PMC6586837; doi:10.1038/s41598-019-45318-5)
Supplement: Supplementary file 1 — Delimiting a species’ geographic range using posterior sampling and computational geometry (Appendices) [file 41598_2019_45318_MOESM1_ESM.docx]

**Running head: delimiting geographic ranges**

Delimiting a species’ geographic range using posterior sampling and computational geometry

(Appendices)

By

Jonathan M. Keith^1,*^

Daniel Spring^2^

Tom Kompas^3^

^1^ School of Mathematical Sciences, Monash University, Clayton, Victoria, 3800, Australia

2. School of Ecosystem and Forest Sciences, The University of Melbourne, 3010, Australia.

3. Centre of Excellence for Biosecurity Risk Analysis, The University of Melbourne, 3010, Australia.

*corresponding author: [jonathan.keith@monash.edu](mailto:jonathan.keith@monash.edu), ORCID iD [0000-0002-9675-3976](http://orcid.org/0000-0002-9675-3976), Phone: +61 3 99020326, Fax: +61 3 99054403

**Appendix 1: Reconstructing the Brisbane fire ant invasion**

**Data**

The data consist of four distinct information types, stored in separate files:

1. **Detections**: the dates and map coordinates (eastings and northings) for each recorded nest detection since the beginning of the eradication program in February 2001, along with the type of detection (public reporting, ground surveillance or aerial surveillance).
2. **Actions**: the grid cell (all cells are 100m by 100m squares) and date of each recorded management action, along with type of action (treatment, ground surveillance or aerial surveillance).
3. **Land use map**: a map indicating which areas in the Brisbane region are urban and which are rural.
4. **Habitat suitability map**: a map indicating for each grid cell the degree of suitability for fire ants (with four categories of suitability, plus a fifth representing water).

A small number of detections with map coordinates corresponding to locations under water in the land use or habitat maps were excluded from all our analyses. The analyses described below were performed for all other detections up to the end of May 2015.

**Model Parameters**

The parameters to be estimated are as follows:

1. **Number of undetected nests**, including those still living, and those killed by treatment without ever being detected.
2. **Location of undetected nests** (the locations of detected nests are of course known).
3. **Month founded** for all nests.
4. **Parent nest** for all nests (the very first introductions do not have a parent nest and are termed “initial nests”, but which nests were original must also be inferred).
5. **Type of founding** for all non-initial nests – this assigns one of four ‘scales’ of founding event, with each scale having a characteristic average distance.
6. **Month killed** for all undetected nests (the month each detected nest was killed is the month it was detected, but undetected nests may be killed by treatments or may still be alive at the end of the period modeled).
7. **Detection probabilities** by the public in urban and rural areas, and by the invasion management agency for ground surveillance and aerial surveillance.
8. **Treatment efficacy** – the probability that a nest in a treated area will be killed by that treatment.
9. **Establishment probabilities** for each habitat suitability type.
10. **Founding type probabilities** – the proportion of founding events in each of the four founding ‘scales’.
11. **Average founding distances** for each of the four founding ‘scales’.
12. **Founding rate** – the average number of nests founded per nest per month, after reaching maturity.

One difference between the model used for this analysis and that described in Keith and Spring^7^ is the explicit consideration of aerial surveillance (also known as *remote sensing*) as one of the survey methods. This method was not considered in our earlier work because it had not been used during the period we analysed (up to 2011). Its extensive use since then was conducted specifically with the aim of achieving delimitation. The inclusion of remote sensing in the model was not difficult: it required only the addition of a remote sensing detection probability.

The model takes into account spatial variation in the probability that citizen monitoring will detect colonies. Urban areas have more people than rural areas, which increases the probability that fire ants will be discovered by citizens in urban areas. For this reason, we constrained the public detection probabilities in urban areas to be greater than in rural areas. We also constrained the probability of detection using ground surveillance by Biosecurity Queensland Control Centre to be at least 0.8 based on unpublished trials conducted in Taiwan by staff of the Biosecurity Queensland Control Centre. We similarly constrained treatment mortality to be at least 0.8 based on an estimate provided in Barr *et al.*^1^ that broadcast bait is likely to result in 80 to 95 percent mortality over a period of 3 to 12 months. Other supporting evidence on which our treatment mortality estimates were based included a recent review by Biosecurity Queensland of treatment methods^2^, which determined that average mortality rates for the treatments used are 83% for methoprene and 95% for pyriproxyfen. We also constrained founding distances to satisfy empirical findings supplied by Biosecurity Queensland, that 90% of alates fly <500m and 99% of alates fly < 2km. These values are supported in the literature^3,4^. However, we found that these values were roughly consistent with what we could infer without enforcing these constraints. Note constraints such as those mentioned in this paragraph are a form of prior information, and were implemented by assigning a prior probability of 0 to regions of parameter space in which the constraints are violated.

Detection by remote sensing is a new monitoring method and its efficacy is less well known than that of ground surveillance. To address this uncertainty we experimented with two plausible alternative estimates of surveillance sensitivity, 0.3 and 0.4, based on advice from Biosecurity Queensland. We also included a lower probability of 0.2 to test the robustness of the inference. The invasion boundaries estimated with this lower remote sensing detection probability are, therefore, outside the most likely boundaries. The two most likely estimates of the remote sensing detection probability were obtained as follows.

1. The remote sensing detection algorithm used by Biosecurity Queensland was tuned to balance detection rate with total output using imagery of known fire ant mounds. This tuning resulted in a detection rate of 40.3%.
2. The detection rate of manual analysis staff was tested consistently by seeding images of known mounds into the manual analysis process. A weighted average was taken that demonstrated manual analysis staff successfully identify fire ant mounds 83% of the time.
3. Allowing for this imperfect detection rate of manual analysis staff reduced the overall detection rate to 33.45% (i.e., 0.403 × 0.83).
4. Our consideration of detection rates of 0.3 and 0.4 reflects that there is a moderate degree of uncertainty about this rate.

The rate at which new colonies are founded is another parameter that is only approximately known, so we trialed several alternative values: 0.15, 0.2, 0.25, 0.3 and 0.35 nests founded per nest per month. These span the range of 2-4 nests founded per nest per year proposed by Biosecurity Queensland, based on unpublished genetic data obtained from the 2013 RIFA incursion at Yarwun in central Queensland.

The probability of detection by remote sensing and the founding rate could instead have been handled by setting prior probabilities to be 0 outside the ranges [0.2,0.4] and [0.15,0.35] respectively. We experimented with this approach during the preparation of this manuscript. We found that this approach estimated the detection probability by remote sensing close to 0.2, at the lower end of the allowed range, and the founding rate close to 0.35, at the upper end of the allowed range. We do not believe these estimates were data driven. Rather, we believe that our model tends to favour scenarios with a larger number of undetected nests. Consequently, where the data provides little or no information about the value of a parameter, the inference is driven by this effect.

The tendency of the model to favour larger numbers of undetected nests could in principle be countered by a suitable choice of prior. However, this seems unnecessary here. Instead, we experimented with different values of these two parameters from across their allowed ranges, in order to determine whether their values affect the inferred geographic range. These investigations are summarised in Figures 5 and 6, and demonstrate that the values of these parameters have minimal impact on the inferred range.

Fire ant colonies at different stages of maturity were explicitly considered to account for differences in their detectability and capacity to reproduce and spread. For example, immature colonies (<8 months) cannot reproduce, and colonies <6 months old are not detectable with the two most commonly used monitoring methods, ground surveillance by trained personnel and aerial surveillance.

**Appendix 2 Simulating a biological invasion**

To illustrate our new method of constructing polygons, we reused a simulated data set that we have described in detail elsewhere^7^. Here we present a brief summary of the simulated data, for the reader’s convenience.

The simulation is intended to resemble the Brisbane RIFA invasion. An initial fire ant nest was assigned to an urban location on a map of Brisbane. The map in question assigns one of four land use types to each location in and around Brisbane, with the majority of locations being identified as either “urban” or “rural”. An unhindered expansion period of 61 months was then simulated, with new nests being founded at a constant rate of 0.25 nests per nest per month. Each nest had a maturation period of 8 months, during which it was unable to found nests. The distribution of *founding distance* (that is, the distance between a parent and child nest) was a mixture of exponential distributions. Reproduction was treated as asexual, so that each child nest had a single parent nest.

Each founded nest was *established* with a probability specific to the *habitat suitability* at that location. Habitat suitability was determined using a second map of the same region, and nests that did not establish were assumed non-viable. Having different establishment probabilities for different habitats models the tendency of the species to prefer certain habitats.

We then simulated an eradication program lasting an additional 95 months. During this time, reproduction was simulated in the same manner as for the unhindered expansion, but in addition the actions of a management program were simulated. Nests were detected and reported by the public with probability 0.02 in any one month for urban areas, and 0.01 in rural areas. When a public detection occurred, a grid cell with side length 100m containing the detection was searched, as were the surrounding eight grid cells. The probability of detection by these targeted searches was 0.8. The sixteen grid cells surrounding these eight were treated but not searched. The probability of a nest being killed by treatment was also 0.8. Nests could be killed by treatment at any age, but nests aged six months or less were considered undetectable by the public or by targeted search.

**Appendix References**

[1] Barr, C.L. *et al.* Broadcast baits for ﬁre ant control. Texas Cooperative Extension B-6099. Texas A&M University (2002).

[2] McNaught, M.K. *et al.* Effect of broadcast baiting on abundance patterns of red imported fire ants (Hymenoptera: Formicidae) and key local ant genera at long-term monitoring sites in Brisbane, Australia. *Journal of Economic Entomology* **107**, 1307-1315 (2014).

[3] Markin, G. P., Dillier, J. H., Hill, S. O., Blum, M. S. & Hermann, H. R. Nuptial flight and flight ranges of the imported fire ant, Solenopsis saevissima richteri (Hymenoptera: Formicidae). *Journal of the Georgia Entomological Society* **6**, 145-156 (1971).

[4] Vogt, J. T., Appel, A. G. & West, M. S. Flight energetics and dispersal capability of the fire ant, Solenopsis invicta Buren. *Journal of Insect Physiology* **46**, 697-707 (2000).
